# Applying the Discrepancy Consistency Method on CAS-2: Brief Data in a Sample of Greek-Speaking Children

**DOI:** 10.3390/jintelligence12040043

**Published:** 2024-04-05

**Authors:** George K. Georgiou, Sergios C. Sergiou, Charalambos Y. Charalambous

**Affiliations:** 1 Faculty of Education, University of Alberta, Edmonton, AB T6G 2G5, Canada; 2 Department of Primary Education, University of Cyprus, Nicosia 4071, Cyprus; sergiou.sergios@ucy.ac.cy (S.C.S.); charalambous.y.charalambos1@ucy.ac.cy (C.Y.C.)

**Keywords:** intelligence, reading, mathematics, specific learning disabilities, discrepancy consistency method, pattern of strengths and weaknesses

## Abstract

This study aimed to examine whether we could use the discrepancy consistency method on CAS-2: Brief data collected in Cyprus. A total of 438 Grade 6 children (201 boys, 237 girls, *M*_age_ = 135.75 months, *SD* = 4.05 months) from Cyprus were assessed on the Cognitive Assessment System-2: Brief that is used to operationalize four neurocognitive processes, namely Planning, Attention, Simultaneous, and Successive (PASS) processing. They were also assessed on two measures of reading (Wordchains and CBM-Maze) and mathematics (Mathematics Achievement Test and Mathematics Reasoning Test). The results showed that 31.5% of our sample had a PASS disorder, and 8% to 10% of our sample had both a PASS disorder and an academic disorder. These numbers are similar to those reported in previous studies that used DCM in North America and suggest that the method can be used to inform instruction, particularly in places where no screening for learning disabilities is available.

## 1. Introduction

Specific learning disabilities (SLDs), an umbrella term used to describe persistent difficulties in reading, writing, and/or mathematical abilities, is one of the most common afflictions identified in school-age populations with a prevalence rate of 3–10% (e.g., Bosch et al. 2021; Moll et al. 2014; Morsanyi et al. 2018). For over two decades, researchers have used the ability–achievement discrepancy model as a means to identify children with SLDs. Within this model, the identification of a SLD is based on a significant difference between the child’s performance on a measure of intelligence and their performance on a measure of academic achievement (e.g., reading). Most commonly, children with a high IQ score and significantly lower academic achievement (e.g., in reading) would be identified as having a SLD. Although widely used, the ability–achievement discrepancy model has been criticized on several grounds. Firstly, children identified as having reading difficulties made similar gains after receiving reading intervention, irrespective of their IQ score (see Lovett et al. 2017); therefore, gains in reading performance did not appear to depend on IQ. Secondly, children with a high IQ score and significantly lower academic performance in reading or mathematics would be identified as having a SLD, even if their academic performance fell within the average range. Finally, using IQ scores to identify children with a SLD sends the message that IQ is strongly related to children’s reading or mathematics performance, regardless of evidence from meta-analytic studies showing weak to moderate associations between IQ and reading or mathematics (e.g., Lozano-Blasco et al. 2022; Peng et al. 2019). Following the criticism of the ability–achievement discrepancy model, researchers turned to other approaches to aid in the identification of children with a SLD, such as response to intervention (RTI) (see Fletcher et al. 2018 ; Reynolds and Shaywitz 2009) and pattern of strengths and weaknesses (PSW). The PSW approach involves identifying a child’s strengths and weaknesses in cognitive processing and determining whether they are consistent with any academic deficits (see Phipps and Beaujean 2016 for a review). While the approach can be operationalized in various ways, each with its own limitations, the current study chose to employ the Discrepancy Consistency Method (DCM; Naglieri 2000). The DCM was chosen because of its alignment with the PASS theory of intelligence (Das et al. 1994, see below) and the Cognitive Assessment System (CAS; Naglieri et al. 2014), which are the focus of this Special Issue. Researchers have argued that the Cognitive Assessment System is culturally fair (e.g., Holden and Tanenbaum 2023; Naglieri et al. 2007) and that it has an advantage over traditional IQ batteries because it measures children’s “thinking” and not children’s “knowing”, which is the case with other popular assessments of intelligence that incorporate scores in vocabulary and mathematics in the calculation of an overall IQ score.

We intentionally chose to apply the DCM on data collected in Cyprus because (a) CAS and CAS-2 have already been used in previous studies in Cyprus and have been found to correlate well with academic achievement and to differentiate children with and without reading difficulties (e.g., Georgiou et al. 2015; Sergiou et al. 2021, 2023), and (b) intervention programs based on the PASS theory have already been developed and tested with success in Cyprus (Papadopoulos et al. 2004; Papadopoulos and Kendeou 2010). Assuming the DCM approach can be successfully applied to this dataset, teachers and practitioners may gain a valuable tool for identifying children potentially impacted by a SLD and who may benefit from intervention. This, in turn, could facilitate the provision of timely intervention. We want to clarify that in using the DCM with CAS data in Cyprus, our objective is not to replace the role of the school psychologist or other professionals for assessing and/or diagnosing children with SLD. Instead, our goal is to offer teachers a tool that may aid in providing them with valuable information about the cognitive processes they can address in their instructional practices.

## 2. The PASS Theory of Intelligence

According to Luria (1966), human cognition involves three functional units that support four neurocognitive processes: Planning, Attention, Simultaneous, and Successive processing (hence, the acronym PASS). The first functional unit is Attention. In order to perform a task, an individual must pay attention to stimuli, resist distraction, and sustain their attention for the duration of the activity. The second functional unit includes two sub-processes: Simultaneous and Successive processing. Simultaneous processing allows an individual to integrate stimuli and process them as a whole. On the other hand, Successive processing allows an individual to process information sequentially. Finally, the third functional unit is Planning. Planning involves formulating a plan, executing it, and evaluating its effectiveness.

In 1997, Naglieri and Das (1997) developed the Cognitive Assessment System (CAS) battery to assess these four cognitive processes. In 2014, Naglieri et al. (2014) published the second edition of the CAS, with an added brief intelligence scale (CAS-2: Brief). The CAS-2: Brief consists of four subtests (Planned Codes, Expressive Attention, Simultaneous Matrices, and Successive Digits), of which the raw scores can be converted to a standard score with a mean of 100. According to Naglieri et al. (2014), “the CAS-2: Brief was designed to be used whenever a brief, reliable, research-based measure is needed to evaluate general cognitive abilities and examination of variability in PASS scores to determine possible implications for intervention” (p. 5).

To date, several studies around the world have shown that PASS processes correlate well with academic achievement (see, e.g., Georgiou et al. 2020), for evidence from a meta-analysis). In addition, children with reading disabilities have been found to perform worse than their chronological-age controls in Successive and Simultaneous processing (e.g., Keat and Ismail 2010; Wang et al. 2012), and children with math disabilities in Planning and Simultaneous processing (e.g., Cai et al. 2013; Deng et al. 2011). Children with comorbid Reading + ADHD or Mathematics + ADHD disabilities have also been found to struggle with attention (e.g., Iglesias-Sarmiento et al. 2017).

Importantly, intervention programs rooted in the PASS theory, such as the PASS Reading Enhancement Program (PREP) and the Math Modules Training, have been shown to improve children’s reading and mathematics performance (e.g., Deaño et al. 2023; Naglieri and Johnson 2000; Papadopoulos and Kendeou 2010; Papadopoulos et al. 2004). If children with specific cognitive strengths and weaknesses are to benefit from a particular intervention program, identifying their cognitive profiles prior to assigning them to a specific intervention program may increase the likelihood of success. For example, PREP targets Simultaneous and Successive processing, both of which have been linked to word reading difficulties. In a study with Greek-speaking Grade 1 children with reading difficulties, Papadopoulos and Kendeou (2010) showed that the children who received PREP improved significantly from pre-test to post-test on several cognitive-linguistic skills (e.g., phonological awareness, rapid naming, and orthographic knowledge) as well as on reading and spelling, and their improvement was significantly larger than that of a control group.

## 3. The Discrepancy Consistency Method (DCM)

**Figure 1:**
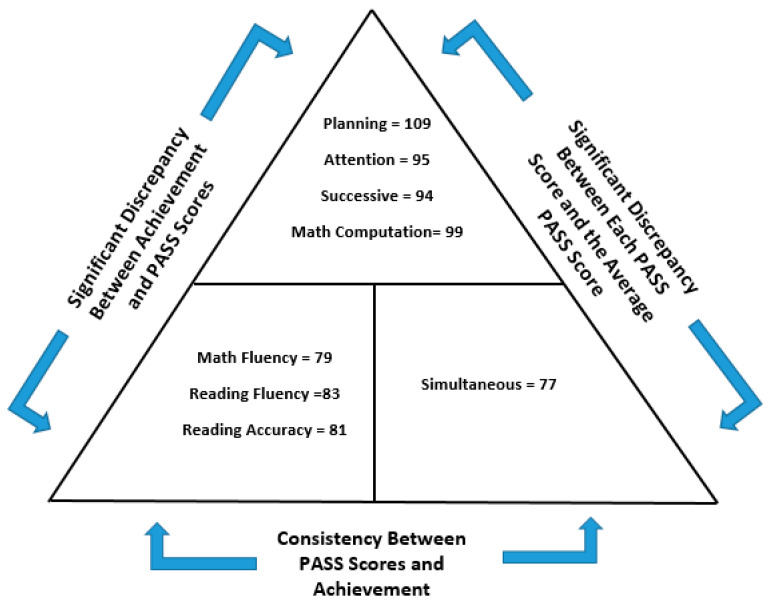
Example of the discrepancy consistency method.

According to Naglieri (2000), (see also Naglieri and Otero 2023), one can use the DCM approach to identify children’s cognitive strengths and weaknesses and subsequently inform instruction. The DCM was originally proposed by Davis (1959) and then further developed by Kaufman (1979) and Silverstein (1993). It is an ipsative method that determines when children’s PASS scores are reliably different from their average PASS score and reliably different from their academic achievement scores. The DCM technique has three parts: two discrepancies and one consistency that jointly form a pattern of strengths and weaknesses (see Figure 1 for an illustration).^1^ A PASS scale discrepancy is found if there is a significant difference among the four PASS scales relative to the child’s overall performance in PASS (e.g., PASS composite score), with one or two PASS scores being substantially below what is considered typical (the normal range). A second discrepancy is found between the PASS strengths and academic weaknesses. The consistency portion of the DCM is found when achievement scores are consistent with the low PASS scores. Such a finding provides evidence that a child has a disorder in the basic psychological processes necessary for a SLD identification (Naglieri and Feifer 2018; Naglieri and Otero 2017).

For example, as shown in Figure 1, the student exhibits strengths in Planning while achieving average scores in Attention and Successive processing. However, the Simultaneous processing score of 77 stands out as notably lower than the average of the four PASS scores (in this case, 93.75). It is crucial to highlight here that strengths in cognition and achievement are evident at the top part of the triangle. Conversely, substantial evidence indicates weaknesses in academic skills, ranging from a mathematics fluency score of 79 to a reading fluency score of 83. These weaknesses align with the Simultaneous processing score of 77, as determined by using the significance values outlined by Naglieri and Otero (2017) and incorporated into the PASS Score Analyzers for all achievement tests using this methodology (Naglieri 2023).

The DCM approach was initially examined by Naglieri (2000) using the standardization sample of CAS that included 1597 children aged 5–18. The results showed that students with a PASS score that was significantly lower than their average PASS score and was below the average range were more likely to experience learning difficulties and need special education. Specifically, he found that 42% of children had relative weaknesses, and 20.9% had cognitive weaknesses. Additionally, 11.8% of children had both cognitive and academic weaknesses. Later on, Huang et al. (2009) examined the profiles of students from the general population (*n* = 1692) and students with an SLD (*n* = 367). They detected 10 core PASS profiles for those children from regular classes and 8 profiles for the students with a SLD. Huang et al. (2009) concluded that their “analysis has provided evidence for the use of the PASS theory and that it appears that it has sufficient applications for diagnosis for students suspected of having an LD” (p. 28). More recently, Georgiou et al. (2022) used the DCM to examine the neurocognitive profile of gifted children. They found that 4% of them had both a PASS disorder and an academic disorder, and they would qualify as twice exceptional (i.e., gifted children who have some form of disability such as SLD). To the best of our knowledge, no studies using the DCM have been conducted with CAS-2, and none of the studies that used DCM included data from outside of North America. Thus, beyond the practical implications of using the DCM in Cyprus, the findings of this study also allow us to establish the external validity of the DCM approach.

## 4. Method

### 4.1. Participants

To recruit our participants, we first sent a letter of information to the families of 535 Grade 6 children attending 25 public elementary schools (10 urban, 13 suburban, and 2 rural) in Cyprus. This initial phase of participant selection excluded any children who had recently immigrated to Cyprus and did not speak or read Greek fluently. A total of 438 Grade 6 children (201 boys, 237 girls, *M*_age_ = 135.75 months, *SD* = 4.05 months) received parental consent and were subsequently invited to participate in testing. Children did not have any known intellectual, emotional, or sensory disabilities (based on school records). Ethics permission to conduct our study was obtained from the National Institutional Review Board (Approval Number 141690).

### 4.2. Materials

**CAS-2: Brief.** The CAS-2: Brief (Naglieri et al. 2014) correlates strongly with CAS-2 (rs higher than 0.70; see Wang et al. 2012) and includes four measures: Planned Codes, Expressive Attention, Simultaneous Matrices, and Successive Digits. Planned Codes are used to assess Planning. Children are required to figure out and continue different patterns within a specified amount of time. The subtest score is based on the combination of time and number of correct answers across the six items. Expressive Attention is used to assess Attention. Children are required to name as fast as possible the color of the ink in which certain color words are printed. The subtest score is based on the combination of time and the number of correct answers. Nonverbal Matrices are used to assess Simultaneous processing. Children are required to select one of six options that best completes a matrix with a missing piece. The subtest score is based on the total number of items correctly answered (max = 44). Finally, Successive Digits are used to assess Successive processing. Children are required to repeat a series of digits (e.g., 9, 2, 6) in the same order they are pronounced by the examiner. We used the same items as in the original task, but with the Greek names of the digits. The subtest score is based on the total number of items correctly repeated. Naglieri et al. (2014) reported coefficient alpha reliabilities higher than 0.86 for the four measures and 0.94 for the composite score. In our sample, Cronbach’s alpha reliabilities were higher than 0.85 for the four measures.

**Mathematics.** We administered two measures of mathematics that have been used in several previous studies in Greek: the Mathematics Achievement Test (MAT; Kyriakides et al. 2019) and the Mathematics Reasoning Test (MRT; Sergiou and Charalambous 2019). MAT includes 13 items (with sub-items) and examines children’s performance in comparing and operating on numbers (including fractions and decimals), converting between different units, interpreting and presenting data, finding the perimeter and area of given shapes, and solving problems. The maximum score in MAT is 43. Cronbach’s alpha reliability in our sample was 0.87. MRT includes 17 items (with sub-items) and measures children’s performance in reasoning with respect to functional thinking and generalized arithmetic. The maximum score in MRT is 46. Cronbach’s alpha reliability in our sample was 0.88. Both MAT and MRT have been used in previous studies and have been found to have good psychometric properties (Kyriakides et al. 2019; Sergiou and Charalambous 2019). In our sample, MAT correlated 0.74 with MRT.

**Reading.** We administered two measures of reading that have been used in several previous studies in Greek: Wordchains (Georgiou et al. 2012) and CBM-Maze (Kendeou et al. 2015). The Wordchains task is similar to the Test of Silent Word Reading Fluency (Mather et al. 2014) and requires children to put slashes to separate as many words as possible that are printed without a space in between them (e.g., πόλημέσατοίχος → πόλη/μέσα/τοίχος). The test had a total of 15 rows of words of increasing length and was discontinued after a minute. A participant’s score was the number of correctly separated words (max = 180) in a 1 min time limit. Cronbach’s alpha reliability in our sample was .89. The CBM-Maze task was adapted in Greek from the work of Deno (1985, 2003). Children were exposed to a 295-word passage in which every 7th word was replaced by three options. The passage was deemed by a group of Grade 6 teachers as appropriate for this grade level. Children were asked to circle the option that correctly completed the meaning of each sentence. A participant’s score was the number of correct answers minus the number of incorrect answers within a 3 min time limit. Cronbach’s alpha reliability in our sample was .89. In our sample, Wordchains correlated 0.69 with CBM-Maze. Previous studies in Greek have also reported construct validity evidence for both Wordchains and CBM-Maze (e.g., Georgiou et al. 2013; Kendeou et al. 2012).

### 4.3. Procedure

All assessments were conducted by trained experimenters in April/May of the school year (about 7 months after the beginning of the school year). The reading and mathematics tests were administered to the whole class, and the CAS-2: Brief was administered to the children individually. The CAS-2: Brief record sheet was translated into Greek by the second author, who is fluent in both English and Greek. In addition, it was back-translated by two professional translators following international standards (Van de Vijver and Hambleton 1996). The protocols were also cross-checked for accuracy of scoring by the second author and another rater, and the inter-rater reliability was 1.00.

### 4.4. Statistical Analyses

Following the instructions provided by Naglieri (1999), we examined the data for four different patterns of strengths and weaknesses. The first two conditions (strength or weakness) were obtained by first computing the differences between each student’s score on each of the CAS:2-Brief measures and that student’s average PASS score (i.e., the composite score) and then comparing those differences to a table of significance provided by Naglieri (1999). This information allowed for the determination of PASS strengths and weaknesses based on what is referred to as an ipsative comparison.

Next, we determined which students in our sample had a significantly low PASS score relative to that student’s average PASS score, and the low PASS score was also below a standard score of 90. This is what is called “PASS Disorder”(Naglieri 1999). The fourth condition examined was a PASS Disorder accompanied by a Reading or Mathematics Deficit based on an achievement score less than *z* = −1.00 in reading or mathematics.

## 5. Results

The descriptive statistics of our measures are presented in Table 1. The variables were normally distributed, and the composite CAS-2: Brief score was close to 100, which means that our sample was behaving similarly to the normative sample. Next, we performed the DCM analysis to identify any students with (a) a PASS weakness, (b) a PASS strength, (c) a PASS disorder, or (d) a PASS disorder with a similar academic skill deficit. The results provided in Table 2 suggest that about 66.6% of the students had a relative strength across the PASS scales and, in most cases, that strength was related to the Planning sub-scale (however, notice that only 22.1% of students were found to have an absolute PASS strength, by obtaining a standard score of 120 or above; Naglieri and Otero 2023). About 45% of the students showed a relative weakness in at least one PASS process. The weaknesses were mainly distributed across the Simultaneous and Successive processing sub-scales. When combined with a PASS score of 85 or lower, these cases indicate a PASS disorder in basic psychological processes. About 31.5% of our sample falls under this category. Interestingly, 9.4% and 8.7% of our sample had a PASS Disorder and low performance in either reading or mathematics, respectively; 4.1% of our sample had a PASS Disorder and low performance in both subject areas.

## 6. Discussion

The overall goal of this study was to examine if we could use the DCM approach in a sample of Greek-speaking children for the purpose of identifying patterns of cognitive strengths and weaknesses that are related to academic achievement. Our motivation in doing so was to examine if the DCM method that has been used in previous studies in North America (e.g., Georgiou et al. 2022; Huang et al. 2009; Naglieri 2000) could provide similar results in a different educational context (i.e., in Cyprus). Applying the DCM method to data collected with CAS-2: Brief was also an important aspect of this study, as we are not aware of a published study that used the DCM with CAS-2. Our findings are very promising as we could identify significant variations in the PASS scores, allowing us to identify which children with a cognitive weakness (i.e., PASS disorder) also had an academic deficit.

More specifically, we found that 31.5% of our sample had a PASS disorder, which is very close to the 29.1% found in the CAS standardization sample (Naglieri 2000). Importantly, this disorder was manifested in Simultaneous and Successive processing, both of which have been linked to reading (Wang et al. 2012) and mathematics (Cai et al. 2013) disabilities. In addition, 8.7% of our sample had both a PASS disorder and a mathematics deficit, and 9.4% had a PASS disorder accompanied by a reading deficit. Acknowledging that the cutoff scores used to select children with reading and/or mathematics deficits play an important role in the prevalence of reading or mathematics disabilities (Koponen et al. 2018), using 1SD below average as our cutoff criterion helped us identify a percentage of children that have frequently been reported in the literature (e.g., Moll et al. 2014; Morsanyi et al. 2018; Yang et al. 2022).

As has been argued before (e.g., Janzen et al. 2013; Maki and Adams 2019; Naglieri and Otero 2017), detecting significant variability in PASS scores is particularly important because it has significant instructional implications. In fact, one of the strengths of using CAS is its direct connection to intervention. The PASS Reading Enhancement Program (PREP) and the Cognitive Enhancement Training (COGENT) were purposely developed to provide teachers with instructional materials that target Simultaneous and Successive Processing. Evidence from studies that used PREP and/or COGENT in different countries, including Cyprus, have produced positive results (e.g., Deaño et al. 2015; Hayward et al. 2007; Mahapatra et al. 2010; Papadopoulos et al. 2004). Similarly, research on Math Modules and the planning facilitation method, both of which were initially designed to improve executive functioning, has produced some positive results (Deaño et al. 2023; Naglieri and Johnson 2000; Iseman and Naglieri 2011). An obvious implication of our findings is that teachers may use the DCM in Cyprus to detect patterns of strengths and weaknesses and then determine what kind of intervention would be more appropriate for certain children.

The present study has some limitations worth noting. First, we intentionally used CAS-2: Brief to provide teachers and practitioners in Cyprus with a viable solution to the problem of using lengthy assessments of cognitive processing. However, CAS-2: Brief includes only one measure of each PASS process. A future study should replicate our findings using the whole CAS-2 battery that includes three measures per scale. Second, our study can be criticized on the same grounds as any other study that uses PSW to identify children with SLD (see Beaujean et al. 2018; Phipps and Beaujean 2016) namely, we assessed children only once, and the prevalence rates may change should we were to retest the same children a few months down the road. However, the same criticism would apply to any other approach to identifying children with a SLD since they rely on assessments conducted once. Third, despite the CAS composite score being 101.59 (thus showing that our sample was performing similarly to the U.S. normative sample), the scores in Planning and Attention were higher than the scores in Simultaneous and Successive processing. Obviously, we do not know if a similar phenomenon was observed in the normative sample, but it is a result that warrants further investigation because, as shown in Table 2, the relative PASS strengths were associated with Planning and Attention, the two processes with high scores. Fourth, we cannot draw any conclusions about the validity of DCM in our sample to identify children with a SLD. Obviously, this would require us to compare if the results of the classification suggested using the DCM method correspond to a diagnosis of a SLD based on established diagnostic criteria or an existing SLD diagnosis, which we did not have. Finally, our study included only Grade 6 children, and our findings may not generalize to other grade levels.

To summarize, our findings add to those of previous studies in which the DCM was applied to data derived from CAS (e.g., Naglieri 2000; Wang et al. 2012) by showing that we can observe significant variation in the PASS scores of our participants and that about 8–10% of them could have both a PASS disorder and a reading or mathematics deficit. Notwithstanding the criticism around the use of PSW for identifying children with SLD (see Phipps and Beaujean 2016), this preliminary evidence can be used to spark future research on identifying patterns of cognitive strengths and weaknesses in Cyprus to guide teachers’ instruction and intervention.

## Figures and Tables

**Table 1:** Descriptive statistics for the measures used in the study.

	Mean	SD	Min	Max	Skewness	Kurtosis
1. Mathematics Achievement Test	27.81	9.83	2	43	0.15	−0.97
2. Mathematics Reasoning Test	20.60	11.45	0	46	−0.56	−0.50
3. WordChains	14.74	5.07	4	36	0.60	0.38
4. CBM-Maze	24.88	7.86	8	46	0.21	−0.35
5. Planning	113.73	10.87	82	145	0.25	0.31
6. Attention	104.03	12.68	72	136	−0.01	−0.08
7. Simultaneous Processing	94.36	10.20	77	121	0.19	−0.72
8. Successive Processing	97.15	9.36	70	118	0.41	0.47
9. CAS-2: Brief Total Score	101.59	11.98	70	135	−0.10	0.22

Note: CAS = Cognitive Assessment System; *N* = 438.

**Table 2:** Percentages of students with significant variability in PASS and achievement test scores (N = 438).

		Planning	Attention	Simultaneous	Successive	
Relative PASS Strength	n	271 (118)	71 (45)	5 (0)	2 (2)	(Unique cases with PASS strength 97–22.1%)
	%	61.8%	16.2%	1.1%	0.4%	Unique cases with relative PASS strength 292–66.6%
Relative PASS Weakness	n	0	12	94	114	Unique cases 195
	%	0%	2.7%	21.4%	26%	44.5%
PASS Disorder	n	0	7	68	59	Unique cases 138
	%	0%	1.6%	15.5%	13.5%	31.5%
PASS Disorder and Mathematics Deficit	n	0	4	19	16	Unique cases 38
	%	0%	0.9%	4.3%	3.7%	8.7%
PASS Disorder and Reading Deficit	n	0	4	21	18	Unique cases 41
	%	0%	0.9%	4.8%	4.1%	9.4%
PASS Disorder and Low Performance in Both Subjects	n	0	2	10	8	Unique cases 18
	%	0%	0.4%	2.3%	1.8%	4.1%

## Data Availability

The data can be made available by sending a request to the corresponding author.

## Note

1 We wish to note here that the pattern of strengths and weaknesses (PSW) approach in identifying children with an SLD is only one of several ways of identifying children with SLD (see Maki and Adams 2019) and that there are mixed opinions regarding its use (see e.g., McGill 2017).
